# Discovery of fragments that target key interactions in the signal recognition particle (SRP) as potential leads for a new class of antibiotics

**DOI:** 10.1371/journal.pone.0200387

**Published:** 2018-07-25

**Authors:** Camilla Faoro, Lorna Wilkinson-White, Ann H. Kwan, Sandro F. Ataide

**Affiliations:** 1 School of Life and Environmental Sciences, The University of Sydney, Sydney, Australia; 2 Sydney Analytical Core Facility, The University of Sydney, Sydney, Australia; University of Queensland, AUSTRALIA

## Abstract

Given the increasing incidence of antibiotic resistance, antibiotics that employ new strategies are urgently needed. Bacterial survival is dependent on proper function of the signal recognition particle (SRP) and its receptor (FtsY). A unique set of interactions in FtsY:SRP-RNA represents a promising candidate for new antibiotic development as no antibiotic targets this complex and these interactions are functionally replaced by protein:protein interactions in eukaryotes. We used a Fragment Based Drug Design (FBDD) approach to search for new compounds that can bind FtsY, and have identified three lead fragments. *In vitro* and *in vivo* analyses have shown that despite a high micromolar binding affinity, one fragment has some antimicrobial properties. X-ray structures of *E*. *coli* FtsY:fragments reveal the fragments bind in the targeted RNA interaction site. Our results show that FBDD is a suitable approach for targeting FtsY:SRP-RNA for antibiotic development and opens the possibility of targeting protein:RNA interactions in general.

## Introduction

Antibiotic resistance represents an increasingly serious threat to global public health. Not only has the discovery of new antibiotics slowed dramatically in the last 20 years, resistance to new drugs is developing rapidly [1]. Traditionally, antibiotic discovery efforts have focused on a few components and pathways in bacteria: namely the bacterial cell wall, the bacterial ribosome and key enzymes involved in the synthesis of essential nutrients. Because new drugs have tended to target the same components and pathways as the parental compounds for which resistance already exists, resistance continues to quickly arise. Therefore, innovative strategies to develop drugs that bind previously untargeted and essential bacterial components are urgently needed.

The signal recognition particle (SRP) is an efficient protein sorting system that directs the translocation of newly synthesized proteins from translating ribosomes to the endoplasmic reticulum (ER) or plasma membrane. SRP recognition helps to circumvent challenges in folding and processing that the nascent polypeptide may face if released into the cytoplasm [2–4]. The sorting of proteins to their appropriate cellular compartment is an essential function, and its disruption is detrimental to all cells [2, 5]. For example, the targeted degradation of SRP triggered protein misdirection and resulted in rapid mitochondrial fragmentation and dysfunction in yeast while truncations or mutations on individual components of the SRP system have proven to be either lethal or severely impact cell viability [5, 6]. However, no known specific inhibitors that target the SRP system have been reported, therefore its targeted disruption may represent a new and promising avenue for antibiotic development.

Despite its high level of functional conservation across all organisms, SRP composition varies considerably in the different domains of life [7–9]. In eukaryotes, the SRP consists of six proteins along with a large RNA moiety, the 7S RNA. In the far simpler version found in bacteria, SRP comprises of one protein (Ffh) and the smaller 4.5S RNA. Similarly, the eukaryotic SRP receptor (SR) consists of two proteins, SRα and the membrane-inserted SRβ, while in bacteria, a single protein, FtsY, is responsible for membrane association and interaction with the translocon [10]. In FtsY, the NG domain (comprising of the N-terminal and GTP binding domains) is involved in binding a GTP molecule and forming a heterodimer with the NG domain of Ffh. However the heterodimerisation along with GTP hydrolysis are mediated by the binding of FtsY to 4.5S RNA, which switches between binding to the RNA tetraloop and the distal region [11–13]. In contrast, these protein:RNA interactions in bacterial FtsY:SRP-RNA are replaced by protein:protein interactions in eukaryotes due to their additional protein components. The importance of biomolecular interactions in SPR:SR systems has been highlighted by a number of complementation studies. In all cases, mutations or deletions in Ffh, FtsY or 4.5S RNA that abolished the aforementioned interactions and/or endogenous activity resulted in cell death or severe impairment [14–16]. Thus, we propose that FtsY:SRP-RNA interactions constitute a highly suitable target for the development of antibiotics.

Traditionally, high-throughput screening (HTS) approaches are favoured in drug discovery projects with millions of compounds screened to identify those that can bind targets with nanomolar affinities [17, 18]. While this approach has produced a number of novel drugs, for example HIV protease inhibitors and imatinib (Gleevec; Novartis), it is generally very costly even when the target structure is well characterised. Fragment-based drug discovery (FBDD) has been proposed as an alternative method that can sample chemical space more efficiently than HTS [17, 18]. The FBDD concept is based on identifying small chemical fragments (typically < 200 Da) with initially weak millimolar affinities to the target site, and then growing or joining them to produce tighter binders. Because of the small size of the fragments, libraries with only a few thousand molecules can sample the chemical space adequately [19]. However, the weak affinity of fragments for their target requires the use of biophysical techniques such as nuclear magnetic resonance (NMR) spectroscopy and/or surface plasmon resonance (SPR) that are highly sensitive for the detection of weak interactions in order to identify initial hits [20].

Here we describe the use of the FBDD approach to identify fragments that bind the NG domain of FtsY (hereinafter called FtsY_NG_). Following initial screens using ligand-based NMR experiments, three fragments from the Monash 2011 library [18] have been shown to induce selective signal changes in the ^15^N-Heteronuclear Single Quantum Coherence (^15^N-HSQC) NMR spectra of ^15^N-FtsY_NG_ indicating binding to specific residues. The dissociation constants of the interactions are in the high micromolar range according to SPR assays, while preliminary zone inhibition assays show at least one of the fragments has some antimicrobial activity. We have also determined the structures of the fragment complexes using X-ray crystallography, which confirmed their binding in the targeted pocket. Our results demonstrate that the FtsY_NG_ and the FtsY:4.5S RNA interactions are suitable targets in an FBDD search for a novel class of antibiotics.

## Materials and methods

### Expression and purification of FtsY_NG_ for ligand-detected NMR experiments, surface plasmon resonance experiments and crystallography

FtsY_NG_ (196–497 aa, corresponding to the NG domain) from *E*. *coli* was inserted into pET15b vector (Novagen) as a fusion protein with an N-terminal 6His-tag [11]. The construct was expressed in *E*. *coli* BL21 cells (DE3) pLysS (Novagen). Freshly transformed competent cells were grown in Luria Broth (LB) media supplemented with 100 μg/mL ampicillin and 50 μg/mL chloramphenicol at 37°C. The preculture was used to inoculate a large volume of LB and cells grown until an OD_600_ of 0.5. Protein expression was induced with 0.5 mM IPTG and cells were grown for another 3 h. Cells were harvested by centrifugation and lysed in lysis buffer (50 mM HEPES, 300 mM NaCl, 10 mM MgCl_2_, 0.1% Triton X-100, 5 mM imidazole, 1 mM TCEP, 1 mM PMSF, pH 7.5) followed by disruption using a French press. The lysate was cleared by centrifugation and loaded onto a column containing Ni-NTA Superflow resin (Qiagen) previously equilibrated with lysis buffer. The column was washed three times with three column volumes of lysis buffer and the protein was eluted with 400 mM imidazole. The eluate was dialyzed overnight at 4°C in dialysis buffer (50 mM MES 100 mM NaCl, 10 mM MgCl_2_, 5% glycerol, pH 6) and loaded onto a HiTrap SP HP cation exchange chromatography column (GE Healthcare) that was equilibrated with buffer A (50 mM MES, 10 mM MgCl_2_, 1 mM TCEP, 5% glycerol, pH 6). The protein eluted within a 30-column volume linear gradient to 50% of buffer B (buffer A with the addition of 1 M NaCl). Fractions containing FtsY_NG_ were pooled and incubated with TEV protease (10,000 U per 25 mg of FtsY_NG_ protein) and dialyzed overnight at 4°C in storage buffer (50 mM HEPES, 300 mM NaCl, 10 mM MgCl_2_, 1 mM TCEP, pH 7.5). The cleaved protein was separated from TEV by Ni-NTA affinity purification. Protein was concentrated with an Amicon Ultra-15 centrifugal filter (Merck Millipore Ltd) to 10 mg/mL and stored at -80°C.

### Expression and purification of FtsY_NG_ for NMR experiments

Uniformly ^15^N-^2^H-labelled FtsY_NG_ was expressed as described in [11]. FtsY_NG_ was purified in a similar way to that described earlier except that the 6His-tag was not removed for NMR experiments.

### NMR spectroscopy

All spectra were recorded on Bruker Avance III 600 or 800 MHz Spectrometers equipped with a TCI cryogenic probehead at 293 K unless otherwise stated.

^1^H one-dimensional (1D) spectra were acquired using IconNMR with the aid of the SampleJet autosampler. Fragments were resuspended in d_6_-DMSO, before being diluted into aqueous buffer as described herein. For the initial cocktail screens, samples consisted of 5 μM FtsY_NG_ in NMR buffer (50 mM sodium phosphate, 100 mM NaCl, 1 mM TCEP in D_2_O, pH 7) with 1 mM fragments. Samples were screened using saturation transfer difference (STD) experiments with the Bruker pulse program stddiffesgp.3 using default settings except for a spinlock time of 20 ms. For individual fragment screens using 1D ^1^H spectra, 400 μM fragment was used in the absence and presence of 10 μM FtsY_NG_. The buffer used was the same as for the initial screens, except that the solvent was 90% H_2_O and 10% D_2_O and with the addition of 1 mM DSS (internal standard). STD experiments on individual fragments were run as described for cocktail screens. All STD spectra are difference spectra and represent the difference between the on and off resonance spectrum for each sample. The % reported in the STD experiment is a numerical representation of the difference in signal intensity between the STD spectrum and the reference spectrum. This % value is then used to rank the binding fragments both in the initial screen and in validation experiments.

Carr-Purcell-Meiboom-Gill (CPMG) experiments were carried out using the modified Bruker pulse sequence cpmges.bm3, which uses excitation sculpting for water suppression and a z-filter to reduce phase errors after spinlock. WATERlogsy experiments were carried out using the pulse program bd_LOGSYesgp.bmlw. This otherwise standard WATERlogsy pulse sequence uses excitation sculpting with gradients for water suppression (similar to the Bruker zgesgp pulse program). Both the CPMG and WATERlogsy pulse programs were obtained from Dr B. Mohanty (Monash University). Samples for ^15^N-TROSY-HSQC were prepared as described for individual 1D ^1^H spectra, with 120 μM FtsY_NG_ and 1 mM fragment. ^15^N-TROSY-HSQC spectra were acquired using the Bruker trosyetf3gpsi pulse program. All NMR data were processed with Topspin 3.5pl7 (Bruker) and mNova (Mestrelab Research).

### Surface plasmon resonance (SPR)

SPR was performed on a Biacore T200. The active and reference flow cells of a Xantec NIHMC Ni sensor chip were conditioned with 0.5 M NaEDTA followed by 5 mM NiCl_2_ in immobilisation buffer (20 mM HEPES, 150 mM NaCl, 3 mM MgCl_2_, pH 7.5). FtsY_NG_ (1 μM) was then injected for 15 min at 10 μL/min over the active flow cell. All immobilisation was carried out at 25 ^o^C. Following immobilisation, the temperature was lowered to 15 ^o^C, and the buffer changed to running buffer (20 mM HEPES, 150 mM NaCl, 3 mM MgCl_2_, 5% glycerol, 5% DMSO, pH 7.5). Samples of fragments (6.25–100 or 200 μM in running buffer) or GMPPNP (6.25–400 μM in running buffer) were injected at a flow rate of 40 μL/min over immobilised FtsY_NG_. Following solvent correction using a DMSO standard curve, equilibrium dissociation constants (K_D_) were calculated by nonlinear least-squares fitting to a simple 1:1 Langmuir binding isotherm, as implemented in the Biacore T200 Evaluation software. Due to the weak nature of the FtsY_NG_:fragment interactions and limited solubility of the fragments, the binding responses for the fragments do not go to completion even at the highest fragment concentrations used. Therefore, the Rmax parameter for the fragment binding was fixed during the curve fitting process and as estimated using the fitted maximum response for the positive control (GMPPNP).

### Crystallography including data collection and refinement

Crystals of purified protein were grown by vapour diffusion using the sitting drop method at 20°C by mixing 400 nL of reservoir solution with 400 nL protein solution (10 mg/mL in storage buffer). Crystals grew within 1 to 3 days in three slightly different conditions (0.1 M Bis-Tris, 28% PEG3350, 165 mM NaCl, pH 6.2) and were harvested after one week of incubation. Prior to flash-freezing, the crystals were cryoprotected by adding well solution containing 25% glycerol. The fragments were soaked at a final concentration of 10 mM on top of already grown crystals for around 1 h prior to freezing.

Diffraction data was collected on the MX1 and MX2 beamlines of the Australian Synchrotron, (Melbourne, Victoria). Diffraction images were indexed using iMOSFLM and data reduction and scaling was processed with the XDS package. The structures were solved by molecular replacement using an in-house model of FtsY_NG_ obtained from an initial phasing with the previously solved FtsY structure (PDB 1FTS) with PHASER (from the CCP4 program suite). Structure refinement was performed with iterative rounds of model building with COOT and model refinement with PHENIX. The bound fragments were allocated in COOT after manual inspection of unmodelled blobs and were refined in PHENIX with ligand restraints generated from eLBOW.

All structures were validated using the PBD Validation Tool and deposited in the protein data bank. The final statistics of the structures are listed in Table A in S1 File. All crystallographic figures were generated using PyMOL.

### Zone inhibition assays

The antimicrobial activity of Fragments 1, 2, and 3 were studied in *E*. *coli* BL21 as the model Gram-negative bacteria and *A*. *baumannii* WM99c as a model for ESKAPE pathogens. Cells were spread evenly on LB agar plates or Sensitest agar plates and then incubated overnight at 37°C in the presence of filter paper discs, previously soaked with fragments (100 or 150 μg per disc). Kanamycin or Amikacin (30 μg) and DMSO were used as positive and negative controls, respectively. The diameter of the inhibition zone was measured after the overnight incubation.

## Results and discussion

### A primary FBDD screen has identified three fragments that bind FtsY_NG_

A key criterion in FBDD library selection is that every library fragment should be amenable to facile synthetic development to facilitate elaboration to more potent compounds. We selected the Monash 2011 fragment library for our primary screen as for most compounds in the library at least 10 related analogues are commercially available. In addition, the library consists of compounds with a variety of characteristics (e.g. pharmacophore fingerprints, shape, physical and chemical properties) and contains 1137 molecules covering all of the two-point pharmacophores and 51% of the three-point pharmacophores [18].

**Fig 1:**
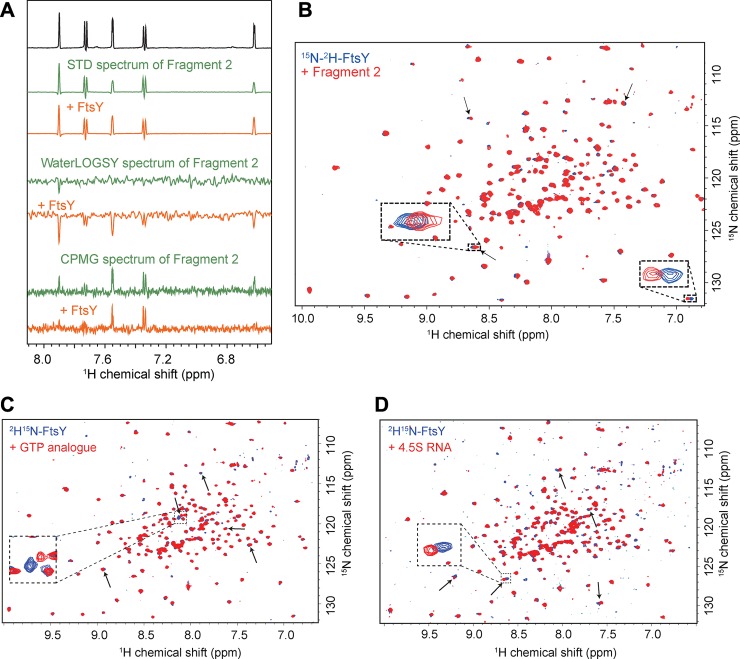
STD, WATERlogsy and CPMG ligand-detected experiments carried out on Fragment 2 in the absence (green traces) and presence (orange traces) of FtsY_NG_. The top (black) trace is the ^1^H 1D NMR spectrum of Fragment 2 for reference. (B−D) ^15^N-TROSY-HSQC spectrum of ^15^N-FtsY_NG_ alone (blue) and following addition of (B) Fragment 2 (red), (C) GTP analogue (red) and (D) 4.5S RNA (red). Arrows indicate the peaks that have shifted during the titration with green arrows highlighting the same peaks that have shifted in the fragment titration and the 4.5S RNA titration. (E) Chemical structures of Fragments 1, 2 and 3.

In order to search for fragments that bind to FtsY_NG_, we produced unlabelled protein recombinantly in *E*.*coli* and purified it using Ni-affinity and cation exchange chromatography [11]. One-dimensional (1D) ^1^H NMR spectroscopy shows that purified FtsY_NG_ in physiological conditions is well-folded and has signal widths consistent with a 33-kDa protein (Figure A in S1 File). We conducted primary screening using saturation transfer difference (STD) NMR spectra with mixtures of three to five fragments in each sample tube [21]. Fragments with an STD difference >7% in the STD-NMR spectra of the mixture were then retested as single compounds using three ligand-based NMR spectroscopic techniques: STD, Carr-Purcell-Meiboom-Gill sequence (CPMG) and WATERlogsy [18, 21]. These ligand-detected NMR experiments exploit the size difference between the ligands and the target proteins. Each of these experiments was carried out on the ligand in the absence and presence of FtsY_NG_. From these experiments, 18 fragments were selected for follow-up. These fragments were considered a “hit” if two out of three experiments showed a positive result, where the threshold was: an STD difference of >5%, a CPMG decrease of >20%, and a WATERlogsy signal change of >100% upon addition of FtsY_NG_ to each ligand (Fig 1A).

After selecting 18 fragments from the initial screen, we further characterised the protein:fragment interactions using ^15^N-HSQC NMR titration studies. We produced ^15^N-^2^H-labelled FtsY_NG_ back exchanged into H_2_O to undertake Transverse Relaxation-Optimised Spectroscopy (TROSY)-HSQC experiments. The TROSY technique offers improved NMR spectral quality for larger proteins (>~30 kDa, [22]) by reducing linewidths [23]. Indeed, the ^15^N-TROSY-HSQC spectrum of labelled FtsY_NG_ displayed sharp and well-dispersed signals (Fig 1B) suggesting the protein is amenable to chemical shift perturbation studies. In this set of experiments, a 10-fold molar excess of fragment was added to the labelled FtsY_NG_ and any spectral changes upon fragment addition were carefully monitored. Three of the selected fragments (termed Fragments 1, 2 and 3, Fig 1E) induced a combined ^15^N-^1^H shift in peak positions of > 0.05 ppm in several peaks in the spectrum, indicating binding to specific residues (Fig 1B, Figure B in S1 File).

Due to the architecture of FtsY and the presence of two distinct binding sites which bind the 4.5S RNA and the cofactor GTP, we additionally carried out TROSY-HSQC titration studies with the 4.5S RNA as well as with a GTP analogue (GMPPNP, 588 Da) [11, 24]. Indeed, two non-overlapping sets of peaks were observed to move in the two ^15^N-TROSY-HSQC titrations confirming the distinct binding sites (Fig 1C and 1D). Encouragingly, some of the FtsY_NG_ peaks that experienced the largest positional changes upon fragment addition correspond to a subset of the peaks that moved in the 4.5S RNA titration (green arrows in Fig 1B–1D, black arrows in Figure B in S1 File). This suggested that the selected fragments and 4.5S RNA are likely to bind overlapping regions on FtsY_NG_.

### Fragments bind FtsY_NG_ with high μM affinity and one has antimicrobial activity

**Fig 2:**
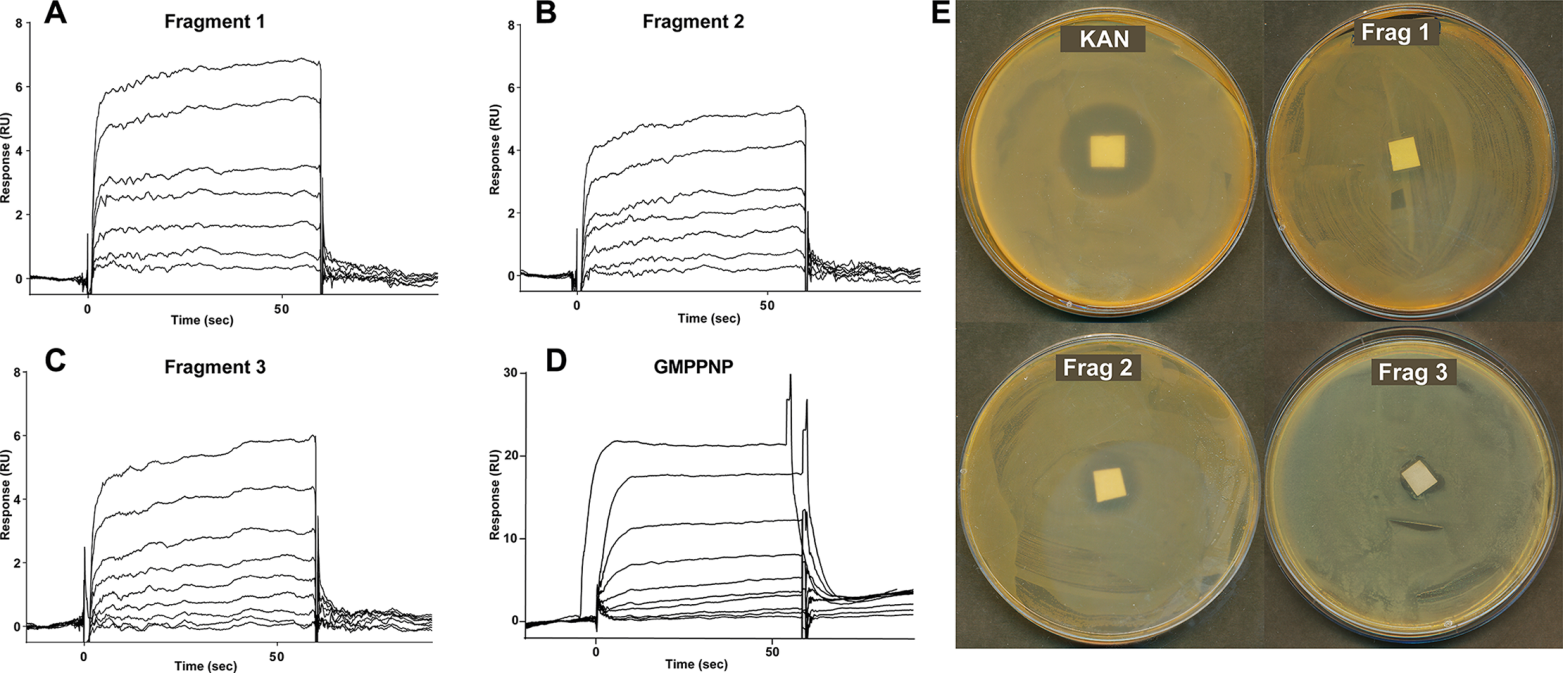
*In vitro* and *in vivo* validation of fragments. SPR measurements performed after immobilization of FtsY_NG_ in sensor chip and Fragment 1 (A), Fragment 2 (B) and Fragment 3 (C) were injected at (6.25–200 μM). As a positive control GMPPNP (D) was injected (6.25 μM–400 μM) and their K_D_ determined. E) Zone inhibition assays using *E*.*coli* BL21were performed with 150 μg of Fragment 1, 2 or 3 spotted in a filter paper prior to overnight incubation at 37°C. Kanamycin (30 μg) was used as a positive control.

Surface plasmon resonance (SPR) is a powerful and adaptable technique for the study of biomolecular interactions, and can measure both the affinity and kinetics of an interaction. SPR has been widely applied as a technique for both primary fragment library screening, as well as for hit validation [25]. The binding affinities for the three fragments that showed a positive result in the ^15^N-TROSY-HSQC experiment, along with the GTP analogue (GMPPNP) were examined by SPR (Fig 2A–2C). The determined K_D_ values between FtsY_NG_ and Fragment 1, Fragment 2 and Fragment 3 are ~100 ± 20, ~100 ± 20 and ~480 ± 20 μM, respectively, while that for GMPPNP is 280 ± 80 μM. The relatively weak binding affinities of the fragments are expected and agree with the small peak positional changes observed in the ^15^N-TROSY-HSQC spectra. Fragments from primary FBDD screens typically bind to their targets in the micromolar to millimolar range [26]. For example, in a study of neurotensin I, the compounds identified had K_D_ values between 18 and 400 μM [27].

Next we tested if the fragments have any antimicrobial effects *in vivo* using *E*.*coli* BL21 in a zone inhibition assay. Fragment 2 showed a larger inhibition zone (20 mm) than Fragment 1 (9 mm) and Fragment 3 (10 mm) (Fig 2D). The presence of a distinct aseptic zone of reasonable diameter indicates that Fragment 2 shows some toxicity towards *E*. *coli* BL21, however, the effect is small and only detectable when used at concentrations five times higher than the positive control (Kanamycin). Similar results were observed when we performed a stringent test with *A*.*baumannii* as a representative organism from the ESKAPE pathogen set at fragment concentrations 3.3 times higher than the positive control (Amikacin) (Figure C in S1 File). While the mechanism of zone inhibition remains to be elucidated, the observation of some antimicrobial effect is promising and the potency is expected to increase substantially upon further development of the fragment(s) into higher affinity FtsY_NG_ binders.

### X-ray structures show fragments bind FtsY_NG_ in the targeted RNA binding pocket

To further characterise the interaction between the fragments and FtsY_NG_, we first solved the *E*.*coli* FtsY_NG_ (herein referred to as apo) structure by molecular replacement using pdb ID:1FTS [28]. Apo FtsY_NG_ crystals diffracted to 1.45Å with two copies of the protein in the asymmetrical unit (Table A in S1 File). The structures show the N domain of one monomer packing against the G domain of the other copy in the asymmetric unit. The N domain of FtsY_NG_ consists of a four-helix bundle (αN1−N4), while the G domain adopts a classical Ras GTPase fold in which five conserved motifs (G1−G5) are arranged around the nucleotide-binding site [28, 29]. Also an unique αβαβ domain to the SRP GTPase family named Insertion Box Domain (IBD) is present in the G domains and is involved in 4.5S RNA recognition and interaction with Ffh as previously reported [24, 28].

**Fig 3:**
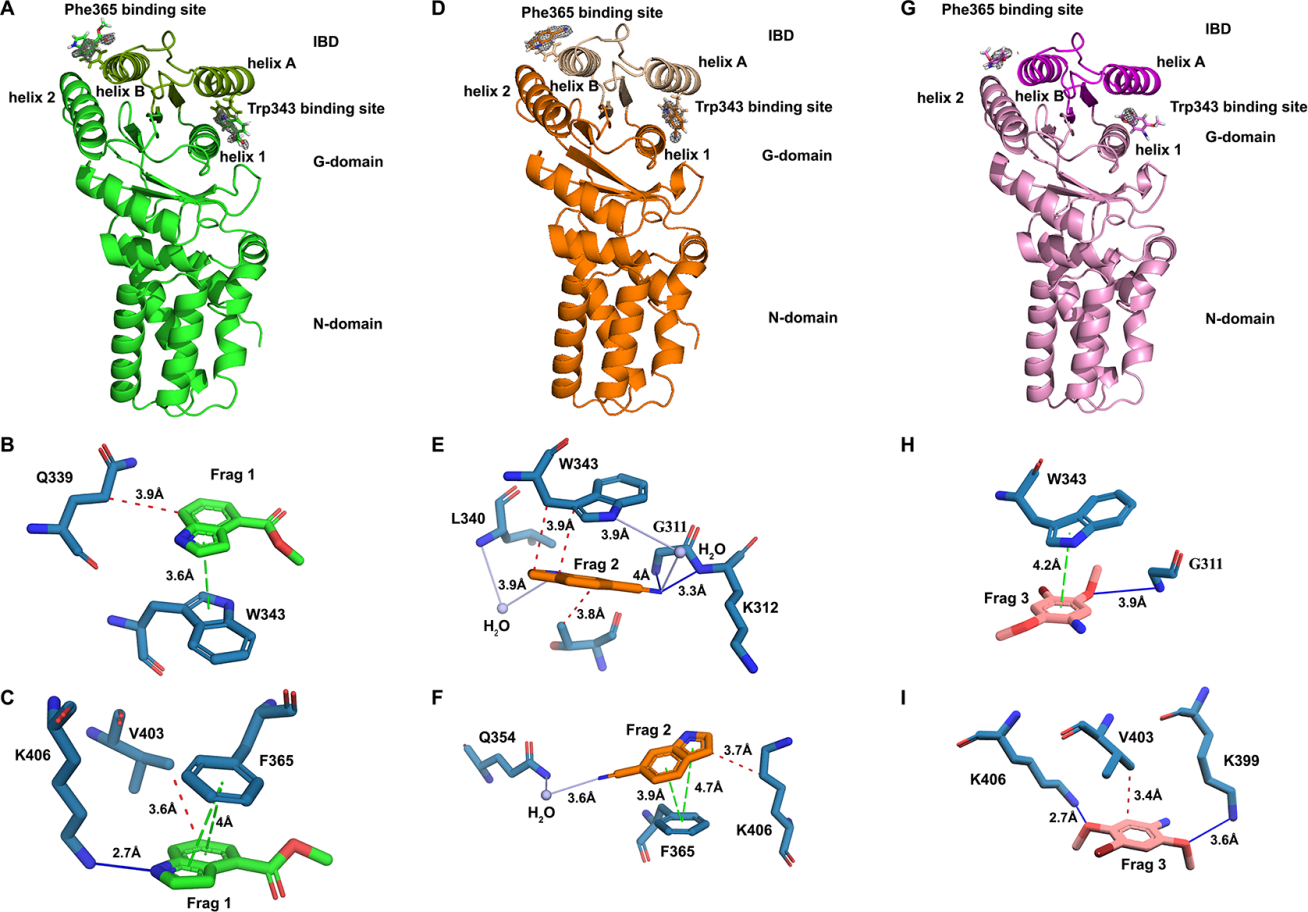
FtsY_NG_ bound with fragments. (A) FtsY_NG_ bound to Fragment 1 (shown in green) in the Trp343 and Phe365 binding sites. (B and C) FtsY_NG_:Fragment 1 interaction profile in (B) Trp343 binding site and (C) Phe365 binding site. (D) FtsY_NG_ bound to Fragment 2 (shown in orange) in the Trp343 and Phe365 binding sites. (E and F) FtsY_NG_:Fragment 2 interaction profile in (E) Trp343 binding site and (F) Phe365 binding site. (G) FtsY_NG_ bound to Fragment 3 (shown in pink) in the Trp343 and Phe365 binding sites. (H and I) FtsY_NG_:Fragment 3 interaction profile in (H) Trp343 binding site and (I) Phe365 binding site. Fragments are displayed as sticks with Fragment 1 shown in green, Fragment 2 in orange, Fragment 3 in pink and the amino acids interacting with them shown as blue sticks. Interactions are water bridge (grey line), hydrophobic (red dashed line), hydrogen bond (blue line) and π-stacking (green dashed line). Distances for interactions are indicated in the figure. mF_o_-DF_c_ fragment electron density map is shown in grey and contoured at 3σ level in (A), (D) and (G).

A 100-molar excess of Fragment 1, 2 or 3 was then added to the drops containing FtsY_NG_ crystals. The soaked crystals were robust and remained stable and diffracted to 1.75−1.85Å. The apo structure was then used as a search model for all subsequent FtsY_NG_:fragment structures (herein referred to as holo structures) using molecular replacement. In the F_o_-F_c_ electron density maps of the holo structures containing Fragment 1, 2 or 3, we observed medium to high densities in the IBD in both copies of FtsY_NG_. At initial refinement steps, the densities were of similar shapes for the three fragments with distinctive features appearing after further rounds of refinement (Figure D in S1 File). Consistent with the relatively low binding affinity observed in SPR experiments, partial occupancies of the fragments yielded maps with lower resolution than expected for a 1.75−1.85Å data set. This indicates a rather large residual mobility of the fragments in the binding pocket. Nevertheless, it is clear that Fragments 1, 2 and 3 bound at two different sites within the IBD in both copies of FtsY_NG_ in the asymmetric unit (Fig 3). The first site is wedged between helix 1 and helix A and involves a stacking with the indole ring of Trp343 (Fig 3). The second site is located between helix B and helix 2 and involves a stacking with the aromatic ring of Phe365 (Fig 3).

Interestingly, the Trp343 site is located between the RNA and GTP-binding sites at the FtsY-Ffh heterodimerization interface (Figure E in S1 File) whereas the Phe365 site is situated in a hydrophobic patch surrounded by charged residues involved in RNA-binding [24] (Figure E in S1 File). Superimposition of the FtsY_NG_:fragment complexes with the previously determined FtsY:Ffh structures bound to the 4.5S RNA at the tetraloop or distal region confirms that the fragments and the Ffh/4.5S RNA bind to an overlapping region (Figure E in S1 File; pdb 4c7o). Therefore, it is expected that fragment binding in either or both of the two sites, Trp343 or Phe365, will likely result in steric hindrance that blocks FtsY:4.5S RNA recognition and prevents heterodimerization with Ffh.

While interactions with Trp343 via π-stacking with the core ring structure are observed in the crystal structures with all three fragments, specific interactions with FtsY_NG_ that are unique to each fragment are also observed. In Fragment 1, hydrophobic interactions involving the CγH_2_ group of Gln339 are also observed whereas in Fragment 2, H-bonds and water bridges with Gly311, Lys312 and Leu340 are seen (Fig 3). For Fragment 3, only H-bonds with Gly311 are additionally present. Given the range of fragment interactions observed, the Trp343 binding site provides a number of options for expanding fragments and exploiting the functional groups of these residues and adjacent ones (Fig 3). Interestingly, Thr307 is also involved with H-bonding to the γ-phosphate of the GTP molecule bound in the GTPase binding site [24] and it is possible that further expansion of the fragment may exploit this property as GTP binding is critical for the function of the SRP complex (Figure E in S1 File). However, GTP binding is highly conserved in both bacterial and eukaryotic SRPs so consideration must be given to potential cross-reactivity if the GTP-binding site is targeted.

In the second (i.e. Phe365) binding site, the three fragments also show the expected interaction with the aromatic ring of Phe365 via π-stacking with the central ring structure. Nonetheless, as for the Trp343 binding site, fragment-specific interactions are also present. Fragment 1 displays hydrophobic interactions with Val403 and H-bonding with Lys406, whereas Fragment 2 forms a water bridge with Gln354 and has hydrophobic interactions with the aliphatic groups of the Lys406 sidechain (Fig 3). In Fragment 3, hydrophobic interactions with Val403 and H-bonding with Lys399 and Lys406 are observed.

**Fig 4:**
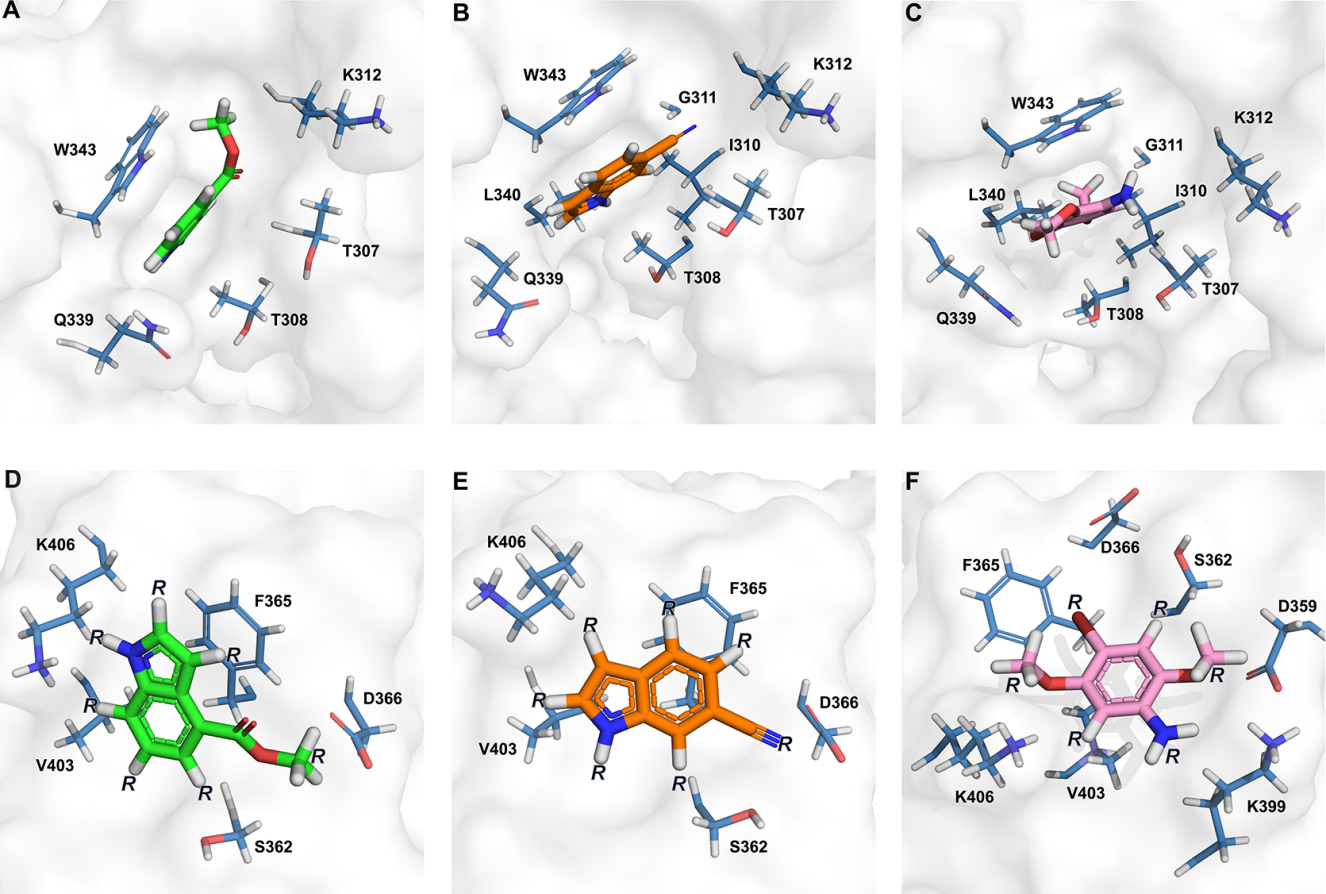
Chemical environment surrounding the fragment-binding sites. In Trp343 binding site: (A) Fragment 1 (green), (B) Fragment 2 (orange) and (C) Fragment 3 (magenta). (D−F) In Phe365 binding site: (D) Fragment 1, (E) Fragment 2 and (F) Fragment 3. FtsY_NG_ is shown as surface with amino acids that form the surrounding fragment binding site labelled and shown as blue sticks. Fragment 1 is shown in green, Fragment 2 in orange and Fragment 3 in magenta. R represents positions to be modified according to the Phe365 binding site.

Reassuringly, the majority of the FtsY_NG_ residues that are involved in fragment interactions, along with Ser362 and Asp366 which are in close proximity, are directly involved in RNA recognition. Identification of both general and specific interactions in the Phe365 and Trp343 binding site gives details of the chemical environment to be exploited in future rounds of drug development to disrupt FtsY_NG_:SRP-RNA interactions. This is currently being explored by delineating the structure-activity relationships (SAR) with the use of fragment analogues (Fig 4) [24]. In particular, we predict substitutions in positions R1, R3 and R5 (from Fragments 1, 2 and 3, respectively, Fig 4D–4F) in the Phe365 binding site with a carboxylic acid or another negatively charged or polar group will build interactions with the positively charged ε-amino group of Lys466. At the other side of the fragments, substitutions with polar or positively charged groups in positions R2, R4 and R6 (from Fragments 1, 2 and 3, respectively, Fig 4D–4F) will be explored to maximise interactions with the negative carboxylic sidechain of Asp366. In addition, a number of other possibilities will be explored to yield analogues that can bind more tightly to the Phe365 binding site.

Here we have reported a primary FBDD screen that led to the identification and characterization of three fragments that can bind the targeted FtsY:RNA binding site with high μM affinity. This suggests that the FtsY:RNA interactions can be targeted by small molecules. In addition, since these interactions are conserved across bacteria and archaea but not in eukaryotes (as the equivalent interactions in the functionally conserved SRP are mediated by additional eukaryotic protein components), the 4.5S RNA binding site in FtsY represents a highly suitable antibiotic target for the design of broad-spectrum antibiotics. Promisingly, we have identified not one but two binding sites within the IBD that are bound by the fragments; interference with either or both of these is likely to disrupt SRP function. This is particularly encouraging and may open a further opportunity to develop a therapy regime where drugs that target each of the binding sites may be combined to achieve a synergistic effect and also help to slow the development of antibiotic resistance, for which multiple and simultaneous mutations by the bacteria would be required.

To our knowledge, this report is the first example of the use of an FBDD screen with NMR spectroscopy and SPR followed by X-ray crystallography structure determination to identify fragments that selectively disrupt protein:RNA interactions. In a post genomic era where the importance of RNAs (both coding and non-coding) and especially their interactions with biomolecules such as proteins are being realised, our work suggests that FBDD may provide an efficient and cost-effective avenue to finding inhibitors that can target other functionally important ribonucleoprotein complexes. Despite the challenge of RNAs being highly negatively charged, RNA interactions can be excellent drug targets as they are essential to the biology/life cycle of microbes and are often unique. In addition, the ability of RNAs to adopt different interaction-dependent 3-D folds may facilitate highly specific drug targeting [30].

From a basic science perspective, the identified fragments may also be used to investigate the molecular mechanisms, including the assembly, interactions, catalytic function and disassembly of the bacterial SRP complex and the roles it plays in protein sorting. While the focus of our investigations has been to find selective inhibitors that only target the FtsY 4.5S RNA binding site, it is foreseeable that the fragments (or more likely their derivatives) can also be tested on the eukaryotic SRP components. This will yield valuable information about the similarities and differences of the two systems. It may also help to shed light on a long-standing conundrum in the field: how RNA-protein interactions in bacteria can be replaced by protein-protein interactions in eukaryotes, and the implications for downstream biological interactions and function.

## Conclusions

In conclusion, the proposed use of the essential SRP complex as a drug target, and the simultaneous targeting of the two unique protein:RNA interaction sites contained therein, represent a significant departure from current strategies for the discovery of antibiotic leads that have focused on the ribosome, cell wall components/machinery and bacterial enzymes. At present, health care-associated infections (HAIs) are a huge issue in the health care industry and a leading cause of morbidity and mortality with an estimated 1.7 million cases and 100,000 deaths annually in the United States alone [31]. It is foreseeable that a new class of antibiotics that target the bacterial SRP through disruption of FtsY interactions will provide a valuable pathway to fighting bacterial infections that are resistant to all currently available drugs.

## Supporting information

S1 File Supporting information for SRP receptor as a novel antibiotic target. (DOCX)
