# Supplementary material for: Discovery of fragments that target key interactions in the signal recognition particle (SRP) as potential leads for a new class of antibiotics
Source: PLoS One. 2018 Jul 25;13(7):e0200387. doi: 10.1371/journal.pone.0200387 (PMC6059433; doi:10.1371/journal.pone.0200387)
Supplement: S1 File — (DOCX) [file pone.0200387.s001.docx]

**S1. Supporting information for SRP receptor as a novel antibiotic target**

**Discovery of fragments that target key interactions in the Signal Recognition Particle (SRP) as potential leads for a new class of antibiotics**

Camilla Faoro^1¶^, Lorna Wilkinson-White^2¶^, Ann H. Kwan^1^*, Sandro F Ataide^1^*

^1^ School of Life and Environmental Sciences, The University of Sydney, Sydney, 2006, Australia

^2^ Sydney Analytical Core Facility, The University of Sydney, Sydney, 2006, Australia

^¶^ These authors contributed equally to this work

* Correspondences to [sandro.ataide@sydney.edu.au](mailto:sandro.ataide@sydney.edu.au) and [ann.kwan@sydney.edu.au](file:///C:\Users\Ann\AppData\Roaming\Microsoft\Word\ann.kwan@sydney.edu.au)

**Table A. Crystallographic statistics**.

| **Structure** | **FtsY apo**  **(6CQP)** | **FtsY-Frag1 (6CVD)** | **FtsY-Frag2 (6CS8)** | **FtsY-Frag3 (6DLX)** |
| --- | --- | --- | --- | --- |
| Space Group | P1 2_1_ 1 | P1 2_1_ 1 | P1 2_1_ 1 | P1 2_1_ 1 |
| Cell dimensions  a, b, c (Å)  α, β, γ (^o^) | 34.7, 76.1, 108.8  90, 93.6, 90 | 34.3, 76.0, 108.0  90, 90.4, 90 | 34.5, 76.2, 108  90, 90.7, 90 | 34.4, 76.0, 107.8  90, 90.4, 90 |
| Resolution (Å) | 44.22 – 1.45 | 36.01 – 1.78 | 35.99 – 1.75 | 43.99 – 1.85 |
| Observed reflections | 459400 | 234475 | 235620 | 92995 |
| Unique reflections | 99964 | 53304 | 56280 | 47444 |
| I/σ (I) | 2.78 | 2.10 | 1.52 | 2.18 |
| Completeness (%) | 99.2% | 99.8% | 99.5% | 99.3% |
| Rmerge (I) | 0.020 | 0.03 | 0.026 | 0.028 |
| Refinement | Phenix | Phenix | Phenix | Phenix |
| Reflections in test set | 4807 | 2586 | 2773 | 2331 |
| R_free_ | 0.174 | 0.251 | 0.221 | 0.249 |
| R_work_ | 0.132 | 0.202 | 0.18 | 0.200 |
| Total number of atoms | 10458 | 10054 | 9843 | 9727 |
| r.m.s.d bonds (Å) | 0.003 | 0.012 | 0.012 | 0.003 |
| r.m.s.d angles (^o^) | 0.64 | 1.13 | 1.03 | 0.55 |
| Ramachandran plot allowed (%) | 1% | 1% | 2% | 2.03% |
| Ramachandran plot outliers (%) | 0 | 0 | 0 | 0 |
| Average B factor (Å^2^) | 23 | 28 | 34 | 35 |

**
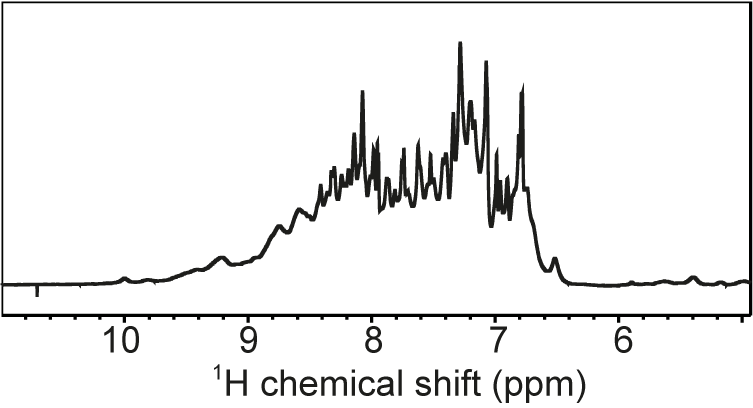
**

**Fig A. Amide region of a 1D ^1^H NMR spectrum of FtsY showing dispersed peaks indicating folded protein.**


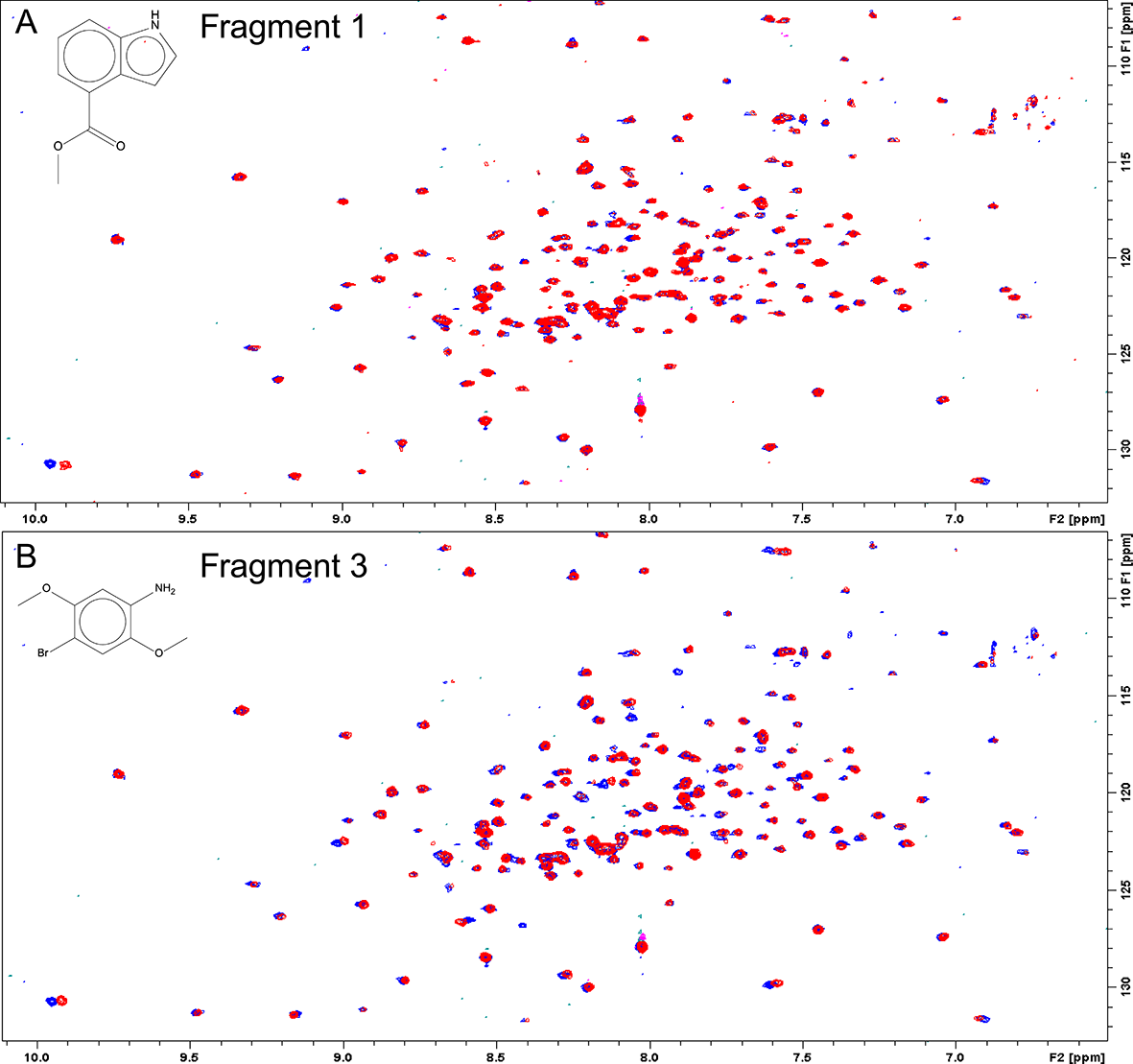


**Fig B. FtsY binding to Fragments 1 and 3.** (A) ^15^N-TROSY HSQC spectrum of ^15^N-FtsY alone (blue) and following addition of Fragment 1 (red). (B) ^15^N-TROSY-HSQC spectrum of ^15^N-FtsY alone (blue) and following addition of Fragment 3 (red). The concentration of ^15^N-FtsY was 120 µM. Arrows indicate some of peaks that have shifted during the titration.


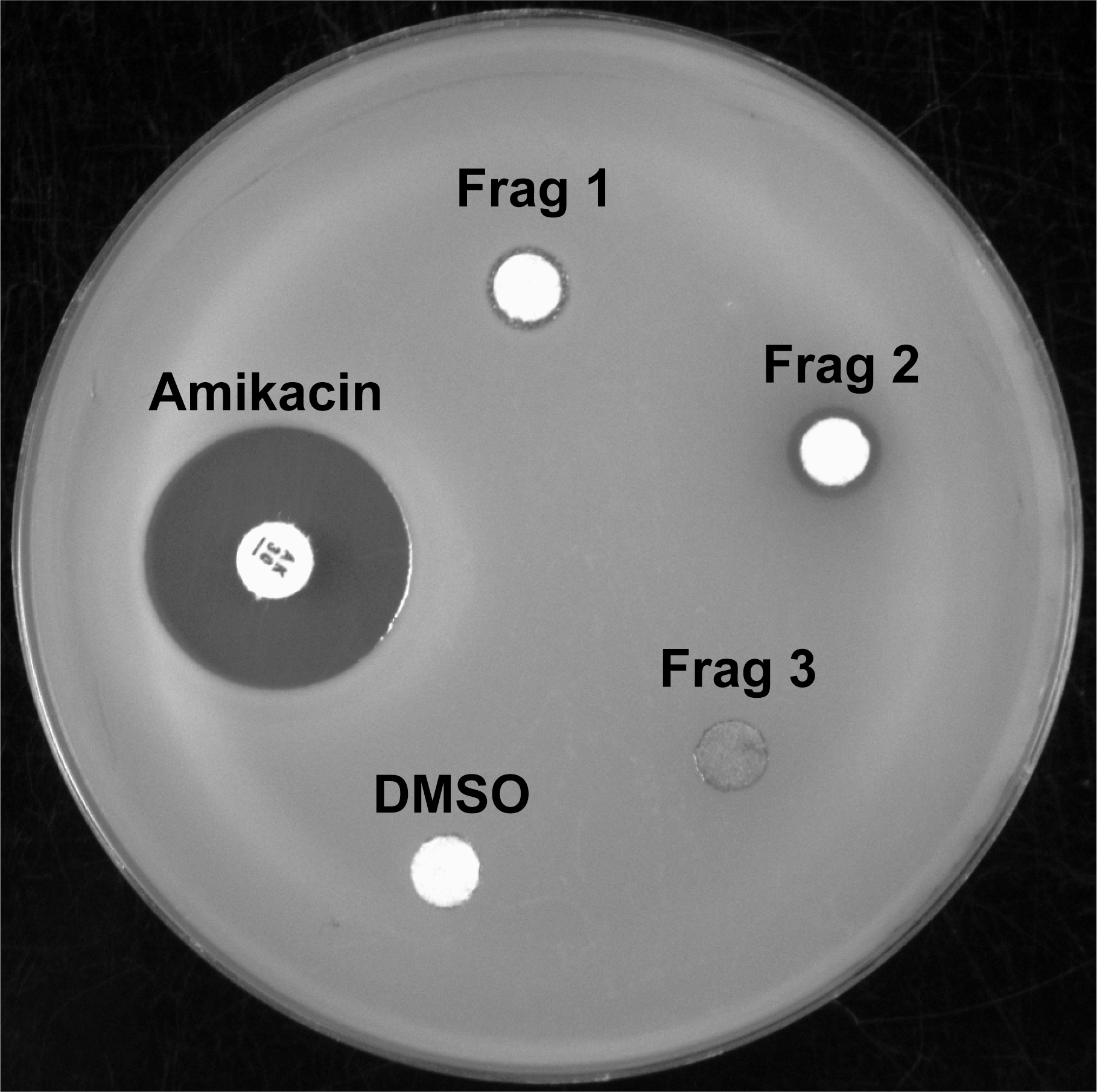


**Fig C**. **Zone inhibition assay with *Acinetobacter baumannii* WM99c strain.** The antimicrobial activities of Fragments 1, 2, and 3 were tested in *A. baumannii* as a representative organism from the ESKAPE pathogen set. Cells were spread evenly on Sensitest agar plates (Oxford, cat# CM409) and then incubated overnight at 37 °C in the presence of filter paper discs previously soaked with Fragments 1, 2 and 3 (100 µg per disc), Amikacin (30 µg) and DMSO were used as positive and negative controls, respectively.


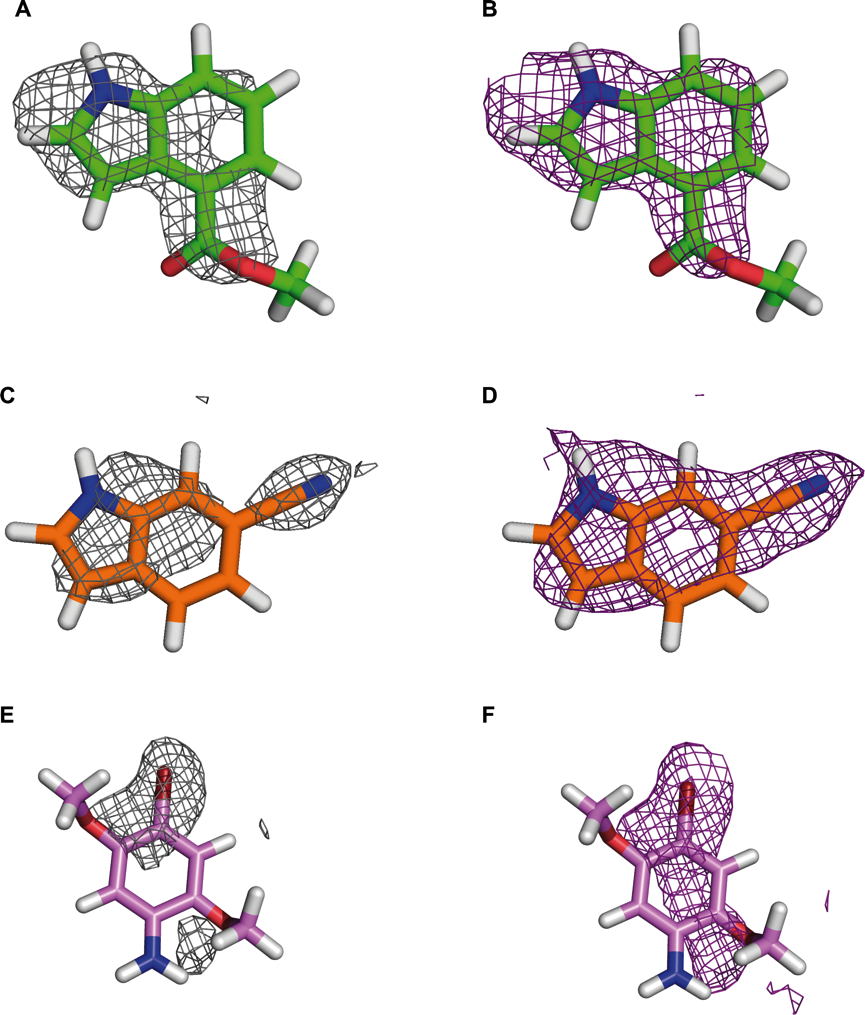


**Fig D. Fo-Fc Fragment electron density map.** (A) Fragment 1 (shown in green) with its omit map (grey) and (B) polder map (magenta) contoured at 3σ. (C) Fragment 2 (shown in orange) with its omit map (grey) and (D) polder map (magenta) contoured at 3σ. (D) Fragment 3 (shown in orange) with its omit map (grey) and (E) polder map (magenta) contoured at 3σ Maps were calculated with Phenix and images rendered in Pymol.


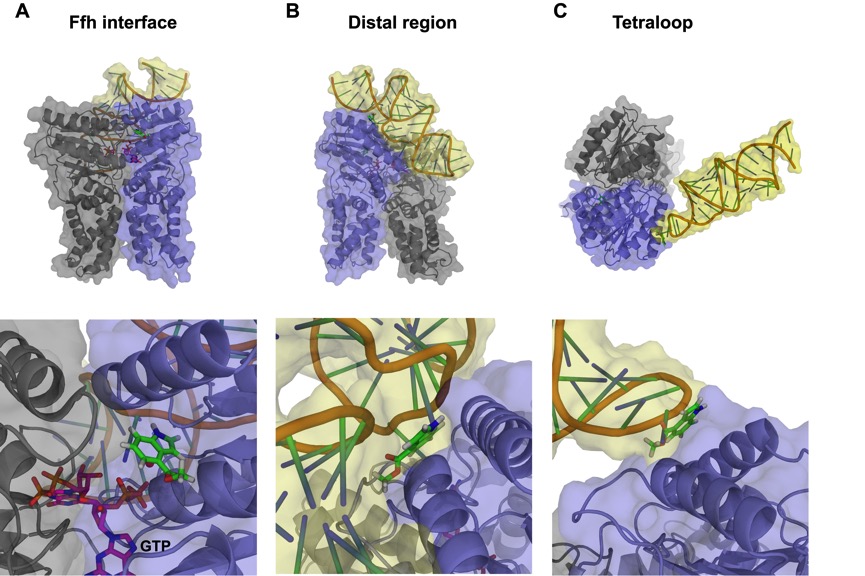


**Fig E**. **Inhibitory mode of fragments on FtsY-SRP interactions.** Superimposition of FtsY bound with Fragment 1 (pdb code 6cvd) and in complex with Ffh and 4.5S RNA fragment (pdb code 4c7o). Each component is represented in cartoon with displayed surface. FtsY is coloured in light blue, Ffh is displayed as grey and the RNA cartoon is coloured by atoms and the surface is shown in yellow. (A) FtsY-Ffh interaction interface. Top panel shows Fragment 1 bound to the Trp343 site that is located next to the GTP binding pocket and between the IBD interactions of FtsY-Ffh. (B) FtsY-Ffh-4.5S RNA Distal region interface. Top panel shows Fragment 1 bound to the Phe365 site and its interference with the binding of the distal region of the 4.5 S RNA. (C) FtsY-Ffh-4.5S RNA tetraloop interface. Top panel shows Fragment 1 bound to the Phe365 site and its interference with the binding of the tetraloop of 4.5 S RNA. All lower panels show close detail of the inhibitory interface.
